# Systematic Elucidation of the Aneuploidy Landscape and Identification of Aneuploidy Driver Genes in Prostate Cancer

**DOI:** 10.3389/fcell.2021.723466

**Published:** 2022-01-21

**Authors:** Yun Peng, Yuxuan Song, Haitao Wang

**Affiliations:** ^1^ Tianjin Institute of Urology, the 2nd Hospital of Tianjin Medical University, Tianjin, China; ^2^ Department of Urology, Peking University People’s Hospital, Beijing, China; ^3^ Department of Oncology, the 2nd Hospital of Tianjin Medical University, Tianjin, China

**Keywords:** prostate cancer, aneuploidy, driver gene, biomarker, prognosis (carcinoma), tumor development and progression

## Abstract

Aneuploidy is widely identified as a remarkable feature of malignancy genomes. Increasing evidences suggested aneuploidy was involved in the progression and metastasis of prostate cancer (PCa). Nevertheless, no comprehensive analysis was conducted in PCa about the effects of aneuploidy on different omics and, especially, about the driver genes of aneuploidy. Here, we validated the association of aneuploidy with the progression and prognosis of PCa and performed a systematic analysis in mutation profile, methylation profile, and gene expression profile, which detailed the molecular process aneuploidy implicated. By multi-omics analysis, we managed to identify 11 potential aneuploidy driver genes (GSTM2, HAAO, C2orf88, CYP27A1, FAXDC2, HFE, C8orf88, GSTP1, EFS, HIF3A, and WFDC2), all of which were related to the development and metastasis of PCa. Meanwhile, we also found aneuploidy and its driver genes were correlated with the immune microenvironment of PCa. Our findings could shed light on the tumorigenesis of PCa and provide a better understanding of the development and metastasis of PCa; additionally, the driver genes could be promising and actionable therapeutic targets pointing to aneuploidy.

## Introduction

Prostate cancer (PCa), the second common male cancer worldwide, led to more than 1.2 million morbidities and 350,000 mortalities in 2018 (Bray et al., 2018; Ferlay et al., 2019). PCa is characterized by the dependent androgen-signaling axis (Dai et al., 2017), driving its progression and proliferation (Mohler, 2008), which introduces the therapies pointing to suppress androgens (Mills, 2014; Watson et al., 2015) and makes antiandrogen therapy [androgen deprivation therapy (ADT)] the main treatment for PCa (Litwin and Tan, 2017). Although PCa is an indolent disease and ADT, abiraterone, enzalutamide (abiraterone and enzalutamide both act on the androgen axis), and chemotherapeutic agent docetaxel have made great advances in treating PCa, the inevitability of eventual castration-resistant prostate cancer (CRPC) makes it remain challenging to treat PCa (Litwin and Tan, 2017; Sartor et al., 2018; Hou et al., 2021; Teyssonneau et al., 2021; Wang et al., 2021; Ziaran, 2021).

Aneuploidy, implying the alteration of the copy number of whole chromosome arms or chromosomes, has been revealed to be a general and remarkable feature of malignancy genomes (Taylor et al., 2018; Zack et al., 2013). Weaver and Cleveland (2006) showed that more than 90% of solid malignancy and more than 80% of hematopoietic carcinoma bore aneuploidy in their genomes. Increasing pieces of evidence suggest that aneuploidy exists more common in tumor genomes than focal copy number alteration (CNA) (Beroukhim et al., 2010) and is implicated in numerous pathways related to genesis, development, proliferation, and metastasis of carcinoma (Taylor et al., 2018; Weaver and Cleveland, 2006; Shukla et al., 2020; Upender et al., 2004). In addition, carcinoma subtypes often present tumor-specific patterns of aneuploidy, and distinctive aneuploidies have been suggested to introduce different drug responses (Shukla et al., 2020; Ried et al., 2012). Moreover, some studies already suggested that aneuploidy induced the aggressive and lethal subtypes of PCa (Ryan and Bose, 2019; Stopsack et al., 2019; Miller et al., 2020; Braun et al., 2013)–(Braun et al., 2013; Ryan and Bose, 2019; Stopsack et al., 2019; Miller et al., 2020).

The overwhelming prevalence of aneuploidy has led to the efforts in the identification of the drivers of aneuploidy, which can help to regard aneuploidy as a potential therapeutic target (Ben-David and Amon, 2020). Several instances suggested that aneuploidy could be induced by some known tumor-associated genes, such as CCND1 (Casimiro and Pestell, 2012), RB1 (van Deursen, 2007), FOXM1, E2F1 (Pfister et al., 2018), and MAD2L1 (Schvartzman et al., 2011), which were mainly implicated in cell cycle pathways for its strong associations with aneuploidy (Hernando et al., 2004; Sotillo et al., 2007). The anomalous expression levels of these aneuploidy driver genes, which may originate from alternative genetic or epigenetic mechanisms including but not limited to point mutations and DNA methylations, contribute to the aneuploidy of malignant carcinoma genome.

Here, we aimed to comprehensively dissect the molecular pathways landscape of aneuploidy and identify potential drivers of aneuploidy in PCa. By providing a perspective to functional pathways across mutation profile, methylation profile, and expression profile, we systematically estimated the molecular processes of aneuploidy in PCa. By combining multi-omics analysis, we tried to identify the potential driver genes of aneuploidy. Because of the connection between aneuploidy and the immune microenvironment (Cristescu et al., 2018; Taylor et al., 2018; Wei et al., 2018), we explored the association of aneuploidy and driver genes with immune infiltration. Finally, we validated these driver genes in Gene Expression Omnibus (GEO) and the Human Protein Atlas and investigated the correlation between driver genes and the special characteristics of PCa in the cBioPortal and GEO database. Our findings could shed light on the effects of aneuploidy on the development and progression of PCa and provide promising therapeutic targets for PCa.

## Materials and Methods

### The Cancer Genome Atlas Prostate Cancer Patient Cohort

The copy number variation (CNV) segmented data of PCa generated by Affymetrix SNP 6.0 platform and DNA copy workflow, the single-nucleotide variation (SNV) data stemming from MuTect2 workflow, the DNA methylation beta value from the platform of Illumina HumanMethylation450, and the raw counts of RNA-sequencing data were downloaded from The Cancer Genome Atlas (TCGA) by TCGAbiolinks (Colaprico et al., 2016) package. Survival-related traits, including both progression-free interval (PFI) and disease-free survival (DFS), and other detailed clinical characteristics of PCa were also derived. PFI and DFS were defined as the interval from diagnosis or from patients' disease-free status after their first diagnosis and therapy, respectively, to the first emergence of a new tumor event (Liu et al., 2018). We used the following criteria to filter samples: firstly, PCa samples must own all data, including CNV, SNV, methylation, and gene expression values; secondly, we only keep PCa samples with overall survival following a time of more than 30 days. Eventually, a total of 459 PCa and 67 normal control samples from TCGA were enrolled in our study. Genes were annotated by the Ensembl database (version 103) (Howe et al., 2021). With regard to DNA methylation profile, CpG probes from SNP, multiple-hit, and allosome were filtered (Zhou et al., 2017). Gene-level methylation values were then defined as the mean methylation values of all CpG probes around transcription start site (interval of not more than 1,000 bp) (Vanderkraats et al., 2013; Teschendorff and Relton, 2018) as previous studies did (West et al., 2013; Jiao et al., 2014). Raw counts of RNA-sequencing were normalized by DESeq2 (Love et al., 2014).

### Genotype-Tissue Expression, Gene Expression Omnibus, and cBioPortal Data Cohort

The raw counts of RNA-sequencing data of 245 normal prostate samples were obtained from GTEx. SU2C dataset (Abida et al., 2019) was downloaded from cBioPortal, including androgen receptor (AR) score and neuroendocrine prostate cancer (NEPC) score (Hieronymus et al., 2006; Beltran et al., 2016; Abida et al., 2019) from 208 PCa samples. GSE21034 (platform: GPL10264; *n* = 179), GSE80609 (platform: GPL11154; *n* = 45), GSE35988 (platform: GPL6480, *n* = 88), and GSE111177 (platform: GPL16791; *n* = 48) datasets were derived from GEO database. Genes in GSE21034 and GSE80609 were annotated by org.Hs.eg.db package (version 3.12.0). GSE35988, with no annotation package in Bioconductor, was annotated by the GEO platform file. GSE111177 was annotated by the Ensembl database (version 103) (Howe et al., 2021).

### Chromosome-Arm-Level Events and Aneuploidy Score

The ABSOLUTE algorithm was applied to determine the purity, ploidy, and absolute copy number. Chromosome-arm-level copy number was determined by weighted median modal copy number (weighted by segment length) across all segmented copy numbers in each chromosome arm, as described in a previous study (Cohen-Sharir et al., 2021). CNA was defined as amplified, neutral, or deleted by comparing the absolute copy number (segment-level or arm-level) with ploidy (rounded to integer number), and segmented copy number spanning the centromere was just split and assigned into respective chromosome arm (Taylor et al., 2018; Cohen-Sharir et al., 2021). Aneuploidy score (AS) was determined by the total altered arms (amplified or deleted) for each PCa sample.

### Other Utilized Scores

Purity standing for the percentage of tumor component was directly derived from the ABSOLUTE algorithm mentioned earlier, and stroma fraction representing the total non-tumor cellular component was estimated by subtracting purity from unity (Taylor et al., 2018; Thorsson et al., 2018). Leukocyte fraction was derived from the study of Thorsson et al. (2018), which defined leukocyte fraction using methylation data. Non-leukocyte stroma fraction was acquired by subtracting leukocyte fraction from stroma fraction (Taylor et al., 2018). TMB was defined as the total number of mutation errors, including base substitutions, insertions, and deletions per megabyte bases (Chan et al., 2019). In our study, the length of exons (38 million) was regarded as the captured gene size. The somatic copy-number alteration (SCNA) score was defined as the total recurrent gene-level CNA determined by GISTIC2.0 (Mermel et al., 2011) (derived directly from TCGA).

### Importance of Aneuploidy to Prostate Cancer

Spearman correlation analysis was utilized to analyze the association of AS with SCNA score or TMB. Univariable Cox analysis and Kaplan–Meier analysis were used to determine the prognostic implications of aneuploidy in PCa. Spearman correlation coefficients, Wilcoxon rank-sum test, and Kruskal–Wallis rank-sum test were utilized to explore the relationships between aneuploidy and clinical characteristics (age, T stage, N stage, M stage, and Gleason score). R package ggplot2 (Wickham, 2016) was used to depict the results.

### Gene Set Enrichment Analysis

As described previously (Taylor et al., 2018), gene set enrichment analysis (GSEA) (Subramanian et al., 2005) was used to determine the hallmark gene pathways in which aneuploidy was most implicated in the MSigDB database (Liberzon et al., 2015) with reference to mutation profile, methylation profile, and gene expression profile. Briefly, genes were ranked decreasingly based on the coefficients of AS from logistic regression (mutation profile) or linear regression (methylation or gene expression profile); ranked genes were forwarded into the GSEA algorithm. By adding purity, leukocyte fraction, or non-leukocyte stroma fraction into the model, we could adjust respective effects on mutation, methylation, or gene expression profiles.

The model equations are as follows (dependent term ∼ independent term):

For mutation profile: logit transformation of gene mutation profile ∼ beta1 * adjusted variable + beta2 * AS.
For methylation or expression profile: genes methylation or expression value ∼ beta1 * adjusted variable + beta2 * AS.

The beta value (beta1 or beta2) represents the coefficients of corresponding variables. The adjusted variable represents purity, leukocyte fraction, or non-leukocyte stroma fraction (one or more).

Bioconductor package clusterProfile (Yu et al., 2012) was applied to conduct the GSEA algorithm. Adjusted *p*-value < 0.05 was regarded as the cutoff. Heatmap was used to delineate GSEA results by ComplexHeatmap (Gu et al., 2016) package.

### Identification of the Aneuploidy Drivers

With regard to mutation profile, gene mutations associated with aneuploidy were identified based on logistic regression; adjusted *p*-value < 0.01 was regarded as the cutoff. For the aspects of methylation and gene expression profile, we conducted both linear regression and Spearman correlation analysis, and only genes meeting this criterion that adjusted *p*-value < 0.01 in linear regression and absolute correlation coefficients > 0.3 and adjusted *p*-value < 0.01 in Spearman correlation analysis were defined as aneuploidy-related genes. The recurrent mutations of PCa were derived from the OncodriveCLUST (Tamborero et al., 2013) algorithm, and the differential methylation genes and differential expression genes were identified by limma (Smyth et al., 2005; Ritchie et al., 2015) and DESeq2 (Love et al., 2014), respectively. As the abnormal expression levels of aneuploidy driver genes may stem from the alternative genetic or epigenetic mechanisms, we could identify potential aneuploidy driver genes by intersecting the gene expression profile produced by aneuploidy-related anomalous mutation profile or gene methylation profile with PCa-specific gene expression. As no recurrent gene mutation was found to associate with aneuploidy and the anomalous expression profile caused by methylation profile is just themselves as the methylation of a gene promoter just regulates the expression of the corresponding gene in reverse directions, we only need to intersect gene methylation profile with PCa-specific gene expression profile, which finally led to the identification of 11 driver genes. Subsequently, GSEA was also applied to determine the associations of driver genes with hallmark gene pathways. A chord diagram by circlize (Gu et al., 2014) package was used to depict the enrichment results.

### Association Between Aneuploidy and Immune Characteristics

Immune subtypes information of PCa and genes coding immunomodulators and chemokines were collected from the study of Thorsson et al. (2018) and the study of Charoentong et al. (2017), respectively. A total of four immune subtypes, including C1 (wound healing), C2 (IFN-gamma dominant), C3 (inflammatory), and C4 (lymphocyte depleted) were found. Kruskal–Wallis rank-sum test was used to grope the associations between immune subtypes and AS or driver genes. Spearman correlation coefficient was further calculated to explore the connections between AS and immunomodulators or chemokine. CIBERSORT (Newman et al., 2015; Chen et al., 2018) algorithm was used to enumerate the infiltration levels of 22 sorts of immune cells based on transcript per million values estimated from fragments per kilobase of exon per million mapped fragments values downloaded from TCGA database (Li and Dewey, 2011). Spearman correlation analysis was further conducted to explore the associations of aneuploidy and its driver genes with the infiltration levels of 22 types of immune cells.

### Determination of the Biological Function of Driver Genes

Gene Ontology with reference to biological process sub-ontologies and Kyoto Encyclopedia of Genes and Genomes were utilized to excavate the underlying molecular function of driver genes in PCa. We also used the Spearman correlation analysis, Wilcoxon rank-sum test, and Kruskal–Wallis rank-sum test to explore the relationships between driver genes and clinical characteristics, including age, T stage, N stage, M stage, and Gleason score.

### Validation of the Importance of Driver Genes to Prostate Cancer

Univariable Cox analysis with reference to PFI or DFS was applied to uncover the prognostic implications of driver genes. GSE21034 was used to confirm the differential expression of driver genes. Moreover, the protein levels of driver genes in PCa and normal prostate were explored in the Human Protein Atlas (Uhlén et al., 2015; Thul and Lindskog, 2018). AR score and NEPC score, the important characteristics of PCa (Hieronymus et al., 2006; Beltran et al., 2016), were further analyzed in the SU2C dataset. We also explored the expression values of driver genes across the varied stage of PCa using the GSE80609 and GSE35988 datasets. Furthermore, the GSE111177 dataset was used to determine the associations of driver genes with ADT.

### Statistical Analysis

All statistical tests were based on a significant *p*-value < 0.05 except for special instructions. The method of Benjamini and Hochberg (1995) was used to adjust the *p*-value when the analysis was involved in multiple comparison problems. We used the R program (version 4.0.5) (ore Team (2020). R: A, 2020) for most of our analysis.

## Results

**FIGURE 1:**
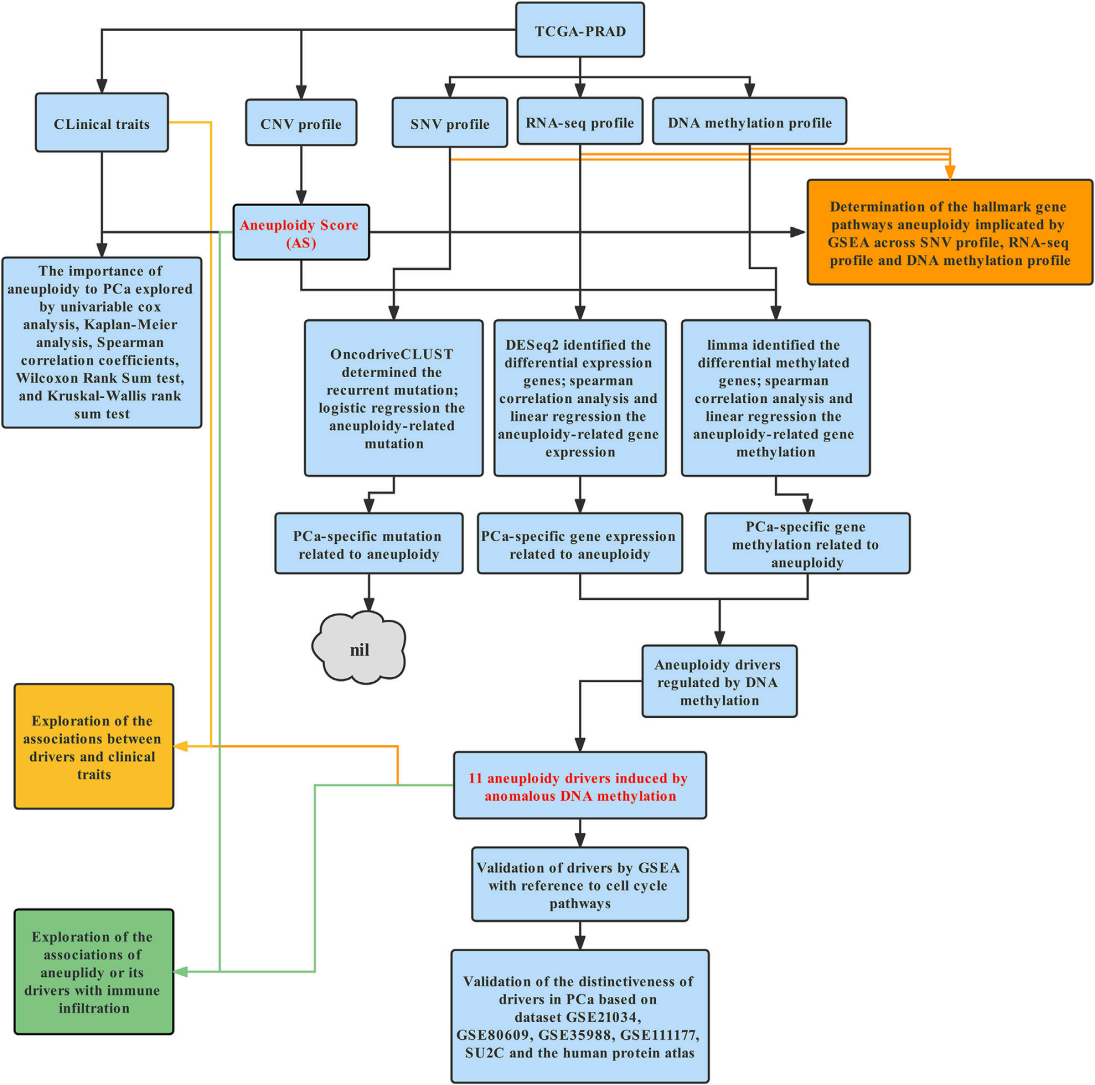
Schematic diagram of this study. Briefly, data including CNV data, SNV data, methylation beta values, and RNA-sequencing data of PCa were downloaded from TCGA. ABSOLUTE was used to estimate AS. GSEA was applied to illustrate molecular process of aneuploidy in mutation, methylation, and expression profile. Next, we tried to identify aneuploidy drivers; as abnormal expression levels of aneuploidy driver genes may stem from alternative genetic or epigenetic mechanisms and genes mainly exert their influence by gene expression, we could identify potential aneuploidy driver genes by intersecting aneuploidy-related mutation profile or gene methylation profile with PCa-specific gene expression. Finally, Considering the close association between aneuploidy and cell cycle pathway, we applied GSEA to validate underlying connection of aneuploidy with driver genes. Further exploration and validation of driver genes were conducted in cBioPortal, GEO, and Human Protein Atlas database.

The schematic diagram for this study is depicted in Figure 1.

### Characteristics of Aneuploidy in Prostate Cancer

**FIGURE 2:**
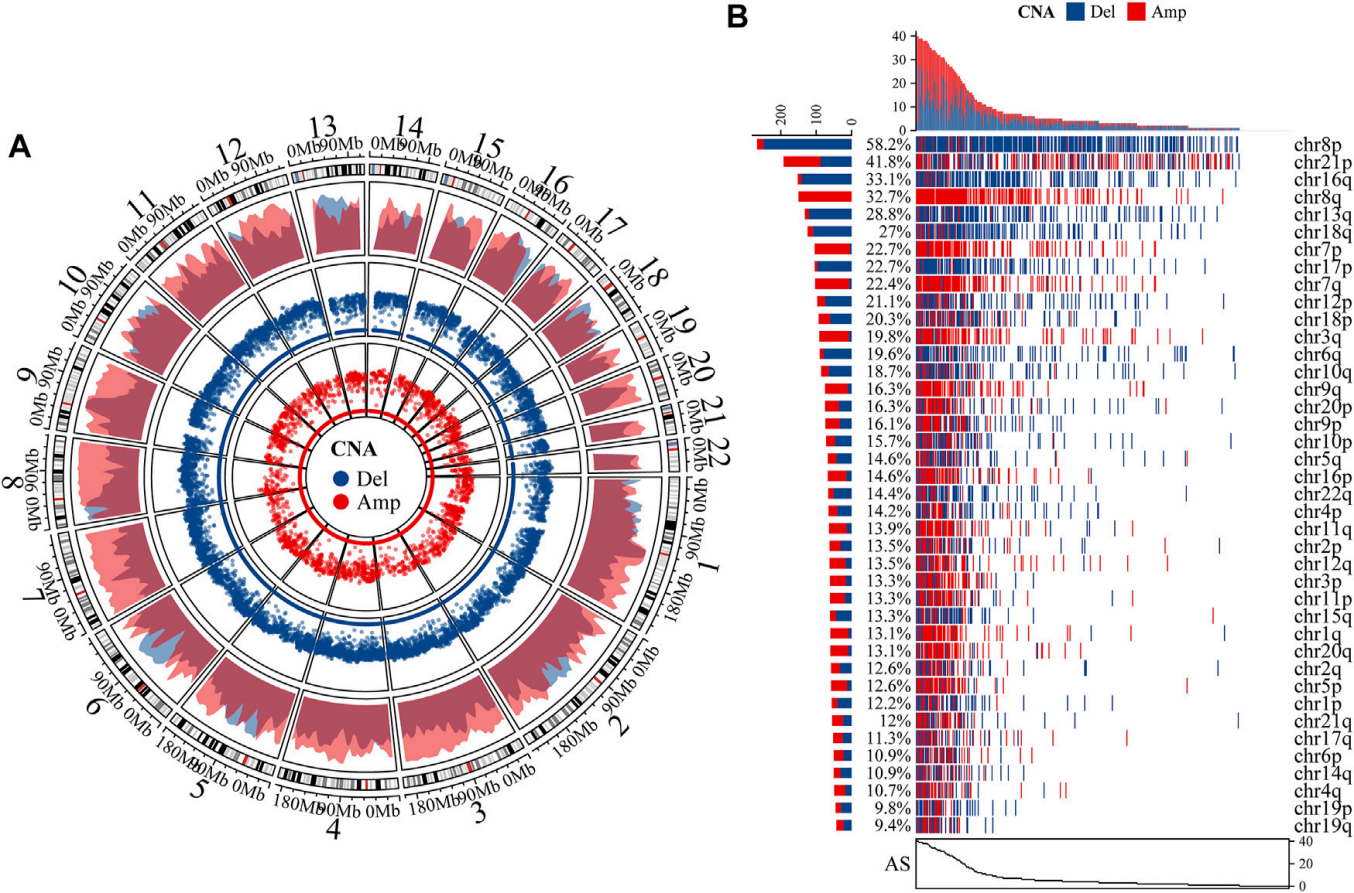
Characteristics of aneuploidy. **(A)** We produced segmented CNA profile by comparing absolute copy numbers with sample ploidy. Outermost ring is autosome ideogram; next is density plot of amplified (Amp) and deleted (Del) CNA whose *y*-axis represents density of regions with CNA. Two innermost rings represent rainfall plot whose *y*-axis stands for log10 (minimal distance of region). Radius of ring represents *y*-axis. **(B)** Waterfall plot of chromosome-arm-level CNA was estimated by comparing weighted copy number with sample ploidy across each chromosome arm.

After comparing the absolute copy number with sample ploidy evaluated by ABSOLUTE, we produced the segmented CNA profile (Figure 2A). Chromosome-arm-level CNAs were further estimated for each chromosome-arm by comparing the weighted copy number with sample ploidy, which showed that the CNAs of chr8p, chr21p, and chr16q accounting for 58.2, 41.8, and 33.1% of PCa, respectively, were the most common aneuploidy (Figure 2B). It seems that chr8p and chr16q often bear deleted copy numbers, but chr8q amplified a copy number. By summing up the CNA in chromosome-arm levels, we estimated AS for each PCa sample, which indicated a total of 86.7% samples that experienced aneuploidy (398 samples in 459 PCas) and AS ranged from 0 to 40 (median AS: 3).

### Importance of Aneuploidy to Prostate Cancer

**FIGURE 3:**
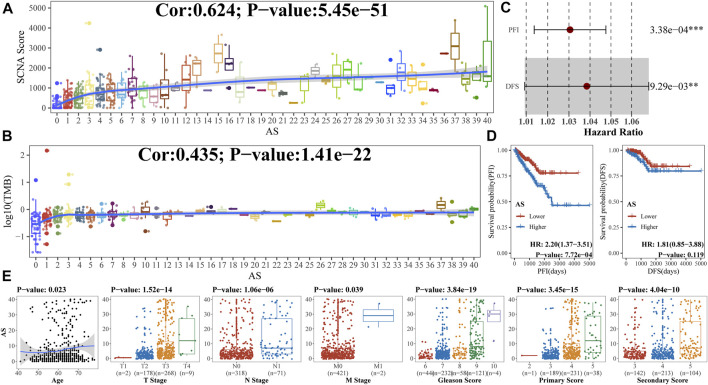
Association of aneuploidy with clinical characteristics **(A, B)**. As revealed in previous study, both SCNA score and TMB were special genomic characteristics and important to carcinoma; we utilized Spearman correlation analysis to uncover relationships between aneuploidy and **(A)** SCNA score or **(B)** log10 (TMB). **(C)** Univariable Cox analysis was applied to confirm prognostic implications of aneuploidy; **(D)** Kaplan–Meier analysis was also used based on median value of AS; **(E)** Spearman correlation coefficients (for age), Wilcoxon rank-sum test (for T stage, N stage, and M stage), and Kruskal–Wallis rank-sum test (for Gleason score) were utilized to explore relationships between aneuploidy and clinical characteristics.

We firstly explored the association by Spearman correlation analysis between AS and some important scores of PCa such as TMB and SCNA score, which indicated that aneuploidy was positively correlated with both (Figures 3A,B). Univariable Cox analysis suggested that aneuploidy was a hazard factor in PCa with reference to PFI [hazard ratio (HR): 1.03 (95% CI 1.01–1.05), *p*-value: 3.38e-04] or DFS [HR: 1.04 (95% CI 1.01–1.07), *p*-value: 9.29e-03] (Figure 3C). Kaplan–Meier analysis gave similar results to aneuploidy with reference to PFI [HR: 2.20 (95% CI 1.37–3.51), *p*-value: 7.72e-04] (Figure 3D). Moreover, we also explored the relationships between clinical characteristics of PCa and AS, which suggested that aneuploidy was associated with tumor progression (Figure 3E; Table 1).

**TABLE 1:** Association of aneuploidy with clinical characteristics in PCa.

	AS				
	level	Overall	Lower	Higher	*p*
n		459	234	225	
Age [mean (SD)]		60.84 (6.84)	60.50 (7.22)	61.19 (6.42)	0.277
T Stage (%)	T1	2 (0.4)	2 (0.9)	0 (0.0)	<0.001
	T2	178 (38.9)	124 (53.2)	54 (24.1)	
	T3	268 (58.6)	104 (44.6)	164 (73.2)	
	T4	9 (2.0)	3 (1.3)	6 (2.7)	
N Stage (%)	N0	318 (81.7)	166 (89.2)	152 (74.9)	<0.001
	N1	71 (18.3)	20 (10.8)	51 (25.1)	
M Stage (%)	M0	421 (99.5)	211 (100.0)	210 (99.1)	0.481
	M1	2 (0.5)	0 (0.0)	2 (0.9)	
Gleason Score (%)	6	44 (9.6)	35 (15.0)	9 (4.0)	<0.001
	7	232 (50.5)	137 (58.5)	95 (42.2)	
	8	58 (12.6)	31 (13.2)	27 (12.0)	
	9	121 (26.4)	31 (13.2)	90 (40.0)	
	10	4 (0.9)	0 (0.0)	4 (1.8)	
Primary Score (%)	2	1 (0.2)	1 (0.4)	0 (0.0)	<0.001
	3	189 (41.2)	127 (54.3)	62 (27.6)	
	4	231 (50.3)	99 (42.3)	132 (58.7)	
	5	38 (8.3)	7 (3.0)	31 (13.8)	
Secondary Score (%)	3	142 (30.9)	85 (36.3)	57 (25.3)	<0.001
	4	213 (46.4)	118 (50.4)	95 (42.2)	
	5	104 (22.7)	31 (13.2)	73 (32.4)	

Group of lower and higher was based on median value of AS. Categorical variables were tested by Fisher's exact test and continuous variables were tested by analysis of variance.

### Landscape of the Functional Pathway Aneuploidy Involved

**FIGURE 4:**
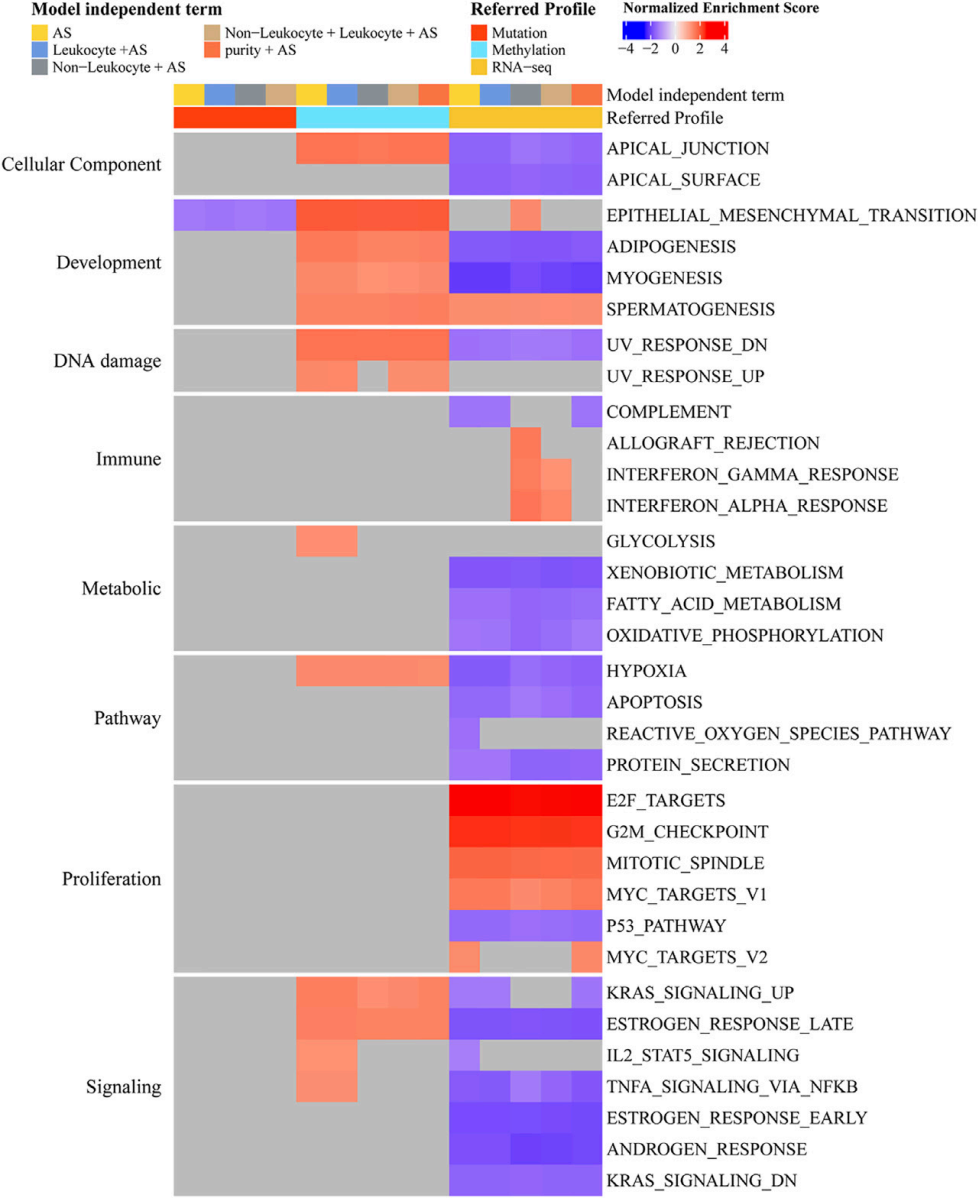
Determination of hallmark function pathway of aneuploidy. Color corresponds to enrichment results of GSEA. Gray means no statistical significance (adjusted *p*-value < 0.05). Genes were ranked decreasingly based on coefficients of AS from logistic regression (mutation profile) or linear regression (methylation or gene expression profile); these ordered genes were further entered into GSEA algorithm. By adding purity, leukocyte fraction, or non-leukocyte stroma fraction into model-independent terms, we could adjust their effects. Model-independent terms describe predictor variables included in logistic or linear regression models.

As aneuploidy was of vital importance to the prognosis and development of PCa, we further analyzed the functional pathway of aneuploidy. From the perspective of the mutation profile in PCa (Supplementary Figures S1, S2), GSEA suggested that aneuploidy was most associated with EPITHELIAL_MESENCHYMAL_TRANSITION (toward the enrichment of mutations among lower-AS samples), even after adjusting to leukocyte fraction or non-leukocyte fraction (Figure 4). With regard to the DNA methylation profile, aneuploidy presented statistical significance in several hallmark gene sets, including APICAL_JUNCTION, all pathways of development, UV_RESPONSE_DN, HYPOXIA, KRAS_SIGNALING_UP, and ESTROGEN_RESPONSE_LATE regardless of the adjustment of purity, leukocyte fraction, or non-leukocyte fraction (toward the enrichment of gene methylation among higher-AS) (Figure 4). From the aspect of the gene expression profile, we observed strong correlations of aneuploidy with proliferation-related pathways (positive association with E2F_TARGETS, G2M_CHECKPOINT, MITOTIC_SPINDLE, and MYC_TARGETS_V1 and a negative correlation with P53_PATHWAY) irrespective of the adjustment of other components (Figure 4). In addition, GSEAs that presented aneuploidy were implicated in varied types of function pathways, including cellular component, development, DNA damage, immune, metabolic, pathway, and signaling subcategory (Figure 4).

### Identification of Driver Genes of Aneuploidy

**FIGURE 5:**
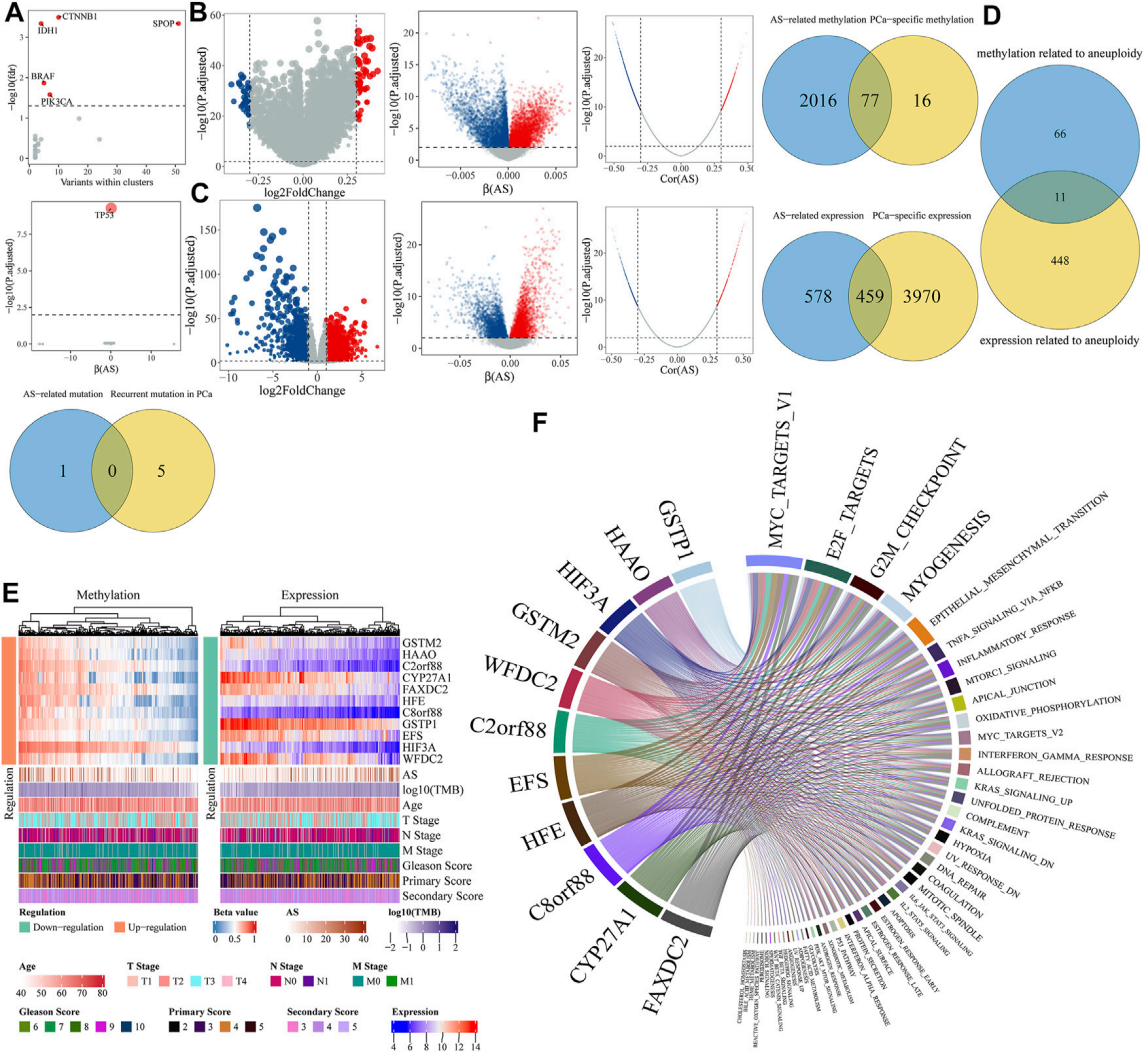
Identification of aneuploidy driver genes. **(A)** Determination of recurrent gene mutations related to aneuploidy. Top panel represents recurrent mutation determined by OncodriveCLUST. Genes with adjusted *p*-value < 0.05 were labeled in red. Middle represents logistic regression results where *x*-axis means coefficient (*β*) of aneuploidy and *y*-axis stands for adjusted *p*-value whose null hypothesis is *β* = 0. Below panel is a Venn diagram by intersecting above two gene sets. **(B,C)** Determination of PCa-specific genes related to aneuploidy in **(B)** methylation profile or **(C)** gene expression profile. From left to right, first panel is a volcano representing PCa-specific genes by differential analysis; second panel represents linear regression results where *x*-axis means coefficients (*β*) of AS and *y*-axis stands for adjusted *p*-value whose null hypothesis is *β* = 0. Red means upregulation, blue means downregulation, and gray means insignificant. Third panel, where *x*-axis means correlation coefficients (Cor) of AS and *y*-axis stands for adjusted *p*-value, represents Spearman correlation analysis results. Red point means positive correlation, blue means negative correlation, and gray means noncorrelation. Genes were regarded as AS-related genes of both |Cor| > 0.3 and adjusted *p*-value < 0.01 in Spearman correlation analysis and adjusted *p*-value < 0.01 in linear regression. Fourth panel is a Venn diagram by overlapping AS-related genes with PCa-specific genes. **(D)** Determination of aneuploidy driver genes regulated by anomalous methylation. **(E)** Heatmap of methylation and expression levels of driver genes. **(F)** Chord diagram illustrates relationships determined by GSEA between driver genes and hallmark pathways.

As the anomaly of driver genes may stem from gene mutation or DNA methylation, we managed to determine driver genes of aneuploidy by multi-omics analysis. In the mutation profile, we identified a recurrent gene mutation in PCa using the OncodriveCLUST (54) algorithm implemented in maftools (Mayakonda et al., 2018) package, which distilled CTNNB1, IDH1, SPOP, BRAF, and PIK3CA with a threshold of adjusted *p*-value < 0.05 (Figure 5A). Meanwhile, logistic regression showed that only TP53 mutation was correlated with AS with a cutoff of adjusted *p*-value < 0.01 (Figure 5A). By intersecting the recurrent gene mutation and aneuploidy-related gene mutation, we got nil of PCa-specific mutations related to aneuploidy (Figure 5A). In the methylation profile, limma showed that 54 genes were up-methylated and 39 genes down-methylated with a threshold of absolute log2FoldChange > 0.3 and adjusted *p*-value < 0.01 (Figure 5B); linear regression and Spearman analysis indicated a total of 2,093 AS-related DNA methylations. Together, we identified 77 PCa-specific gene methylations related to aneuploidy (Figure 5B). As genes mainly executed their influence by gene expression, we also determined the PCa-specific gene expression related to aneuploidy. Considering RNA-seq profile, differential expression analysis revealed 2,145 upregulated and 2,284 downregulated genes with a threshold of absolute log2FoldChange > 1 and adjusted *p*-value < 0.01, meantime, linear regression, and Spearman analysis indicated a total of 1,037 AS-related gene expressions (Figure 5C). Following, we obtained 459 aneuploidy-related gene expressions (Figure 5C). We intersected the key genes related to aneuploidy found in methylation profile with genes in RNA-seq profile, which led to 11 potential driver genes regulated by anomalous DNA methylation in PCa (GSTM2, HAAO, C2orf88, CYP27A1, FAXDC2, HFE, C8orf88, GSTP1, EFS, HIF3A, and WFDC2) (Figure 5D). The DNA methylation value and gene expression value of these genes are depicted in Figure 5E. GSEA showed all driver genes highly referred to the proliferation-related pathway (MYC_TARGETS_V1, E2F_TARGETS, and G2M_CHECKPOINT) (Figure 5F).

### Biological Function and Clinical Correlation of Driver Genes

Gene Ontology and Kyoto Encyclopedia of Genes and Genomes analysis suggested that 11 driver genes were involved in numerous identical processes (noncoding RNA metabolic process, ribosome biogenesis, cytokine−cytokine receptor interaction, RNA transport, ribosome, calcium signaling pathway, herpes simplex virus 1 infection, and so on) (Supplementary Figure S3). We further analyzed the associations of 11 driver genes with clinical characteristics. A slightly negative correlation with the primary Gleason score was observed for all driver genes. In addition, GSTM2 and HFE presented statistical significance to the association with the T stage, and C2orf88, C8orf88, CYP27A1, and EFS were found to be significantly correlated with both the T stage and N stage (Supplementary Figure S4).

### Associations With Immune Infiltration

Kruskal–Wallis rank-sum test suggested aneuploidy, and its 11 driver genes were significantly correlated with immune subtypes (Supplementary Figure S5). To further explore the underlying correlation with immune, Spearman correlation analysis was conducted, which indicated aneuploidy highly referred to the methylation (Supplementary Figure S6A) and expression (Supplementary Figure S6B) of MHC, receptor, chemokine, immunostimulator, and immunoinhibitor. As expected, the methylation levels of most of MHC, receptor, chemokine, immunostimulator, and immunoinhibitor were positively associated with non-leukocyte fraction and negatively with leukocyte fraction (Supplementary Figure S6A), and the expression levels of most of them were contrary to those mentioned earlier (Supplementary Figure S6B). Spearman correlation analysis indicated that aneuploidy was statistically associated with non-leukocyte fraction and stromal fraction but not with leukocyte fraction, which suggested that the observed association of aneuploidy with tumor stromal is much based on a non-leukocyte component in PCa as the previous study has pointed out (Taylor et al., 2018) (Supplementary Figure S6C). Similarly, only a few types of immune cells were found to correlate with aneuploidy (Supplementary Figure S6C). Nevertheless, 11 aneuploidy driver genes showed consistent correlation with immune infiltrated cells (B cells naïve, Plasma cells, T cells CD8, T cells CD4 memory resting, NK cells activated, monocytes, macrophages M2, dendritic cells resting, and Mast cells resting) (Supplementary Figure S6C).

### Validation of Driver Genes

**FIGURE 6:**
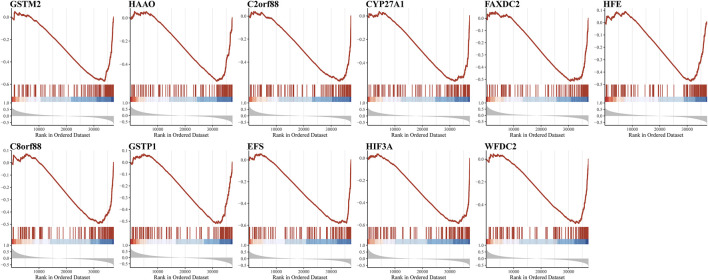
Demonstration of association of aneuploidy driver genes with cell cycle pathways. Because of crucial contributions of cell cycle pathways on aneuploidy, GSEA was utilized to confirm association of aneuploidy driver genes with cell cycle pathways.

**FIGURE 7:**
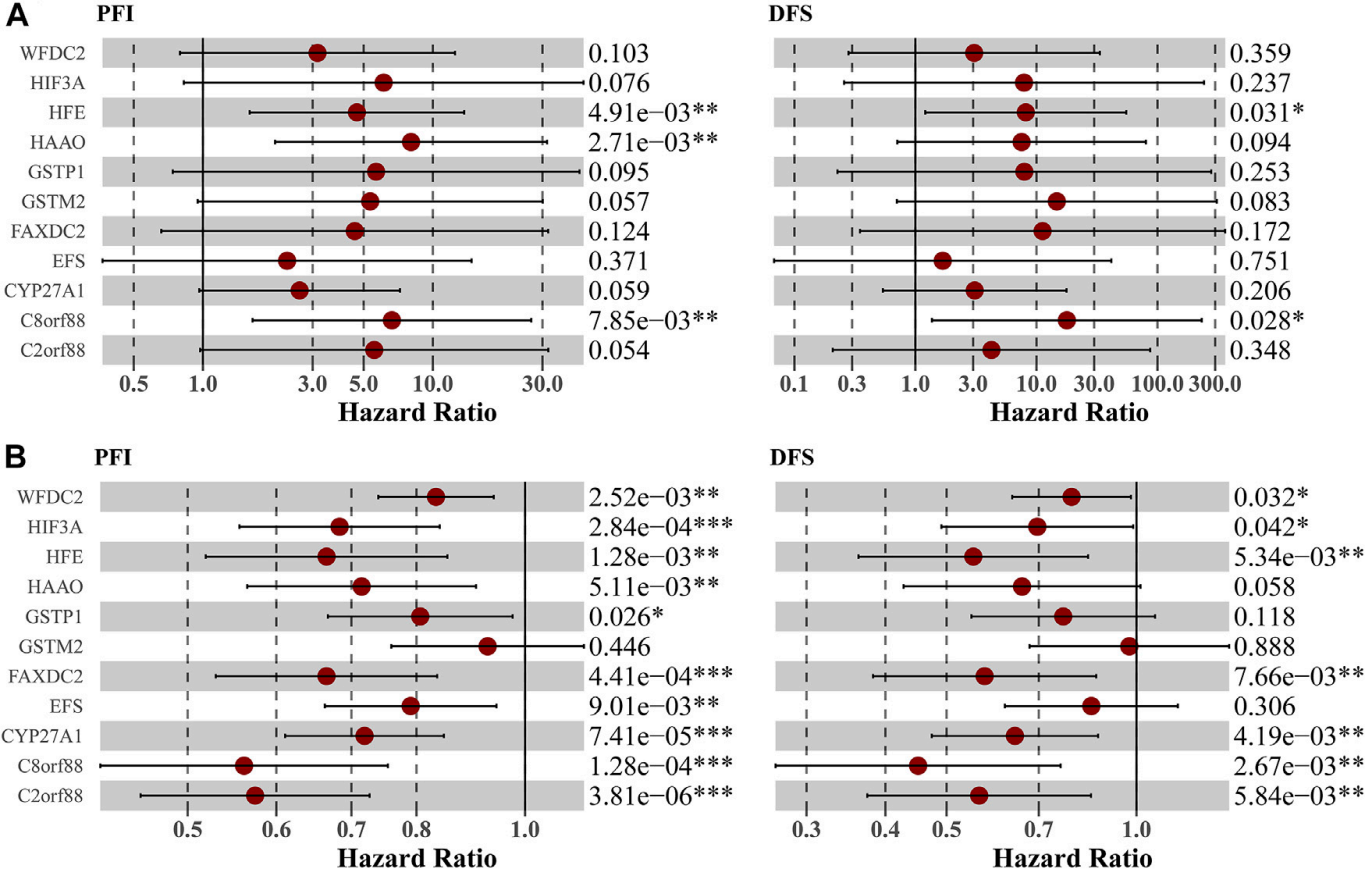
Validation of prognostic implications of driver genes in **(A)** methylation profile and **(B)** expression profile. Univariable Cox analysis was conducted. Label (*) means *p* < 0.05, label (**) means *p* < 0.01, and label (***) means *p* < 0.001.

**FIGURE 8:**
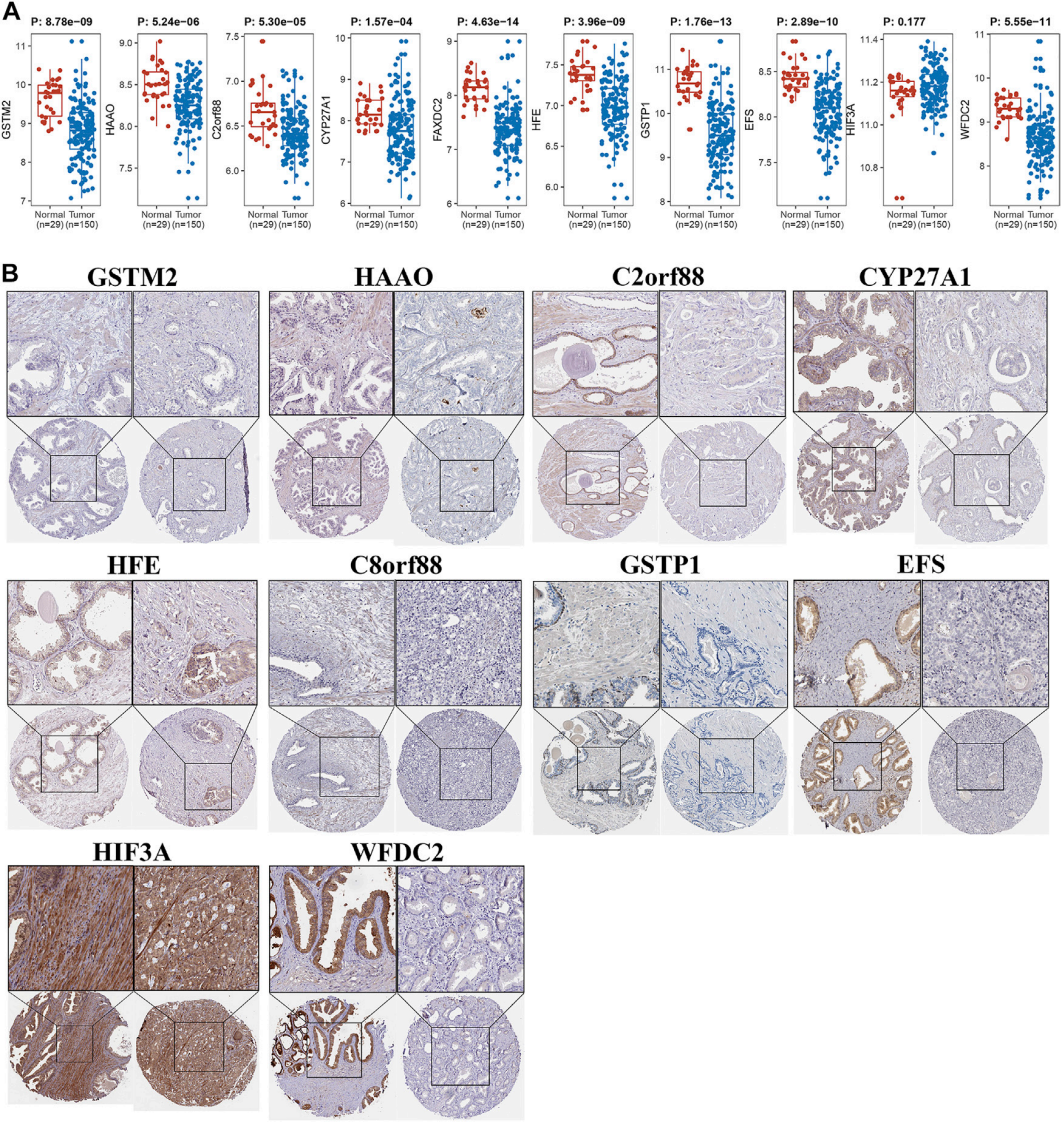
Validation of differential expression of driver genes. **(A)** Wilcoxon rank-sum test was applied to test gene expressional difference of driver genes between PCa and normal control samples. **(B)** Protein levels of driver genes were explored in Human Protein Atlas.

Owing to the close association between aneuploidy and cell cycle pathway, we firstly applied GSEA with reference to cell cycle pathway to confirm the underlying connection between aneuploidy and driver genes, which indicated that all of these 11 driver genes were significantly correlated with cell cycle pathway (Figure 6). To further validate these 11 driver genes, we conducted a univariable Cox analysis. Methylation profile revealed that only HFE, HAAO, and C8orf88 were associated with the prognosis of PCa (Figure 7A); nevertheless, expression profile suggested that the expression of all driver genes except GSTM2 was a protective factor with reference to PFI {GSTM2 [HR: 0.93 (95% CI 0.76–1.13); *p*-value: 0.45], HAAO [HR: 0.71 (95% CI 0.57–0.90); *p*-value: 5.11e-03], C2orf88 [HR: 0.57 (95% CI 0.45–0.73); *p*-value: 3.81e-06], CYP27A1 [HR: 0.72 (95% CI 0.61–0.85); *p*-value: 7.41e-05], FAXDC2 [HR: 0.67 (95% CI 0.53–0.83); *p*-value: 4.41e-04], HFE [HR: 0.67 (95% CI 0.52–0.85); *p*-value: 1.28e-03], C8orf88 [HR: 0.56 (95% CI 0.42–0.75); *p*-value: 1.28e-04], GSTP1 [HR: 0.81 (95% CI 0.67–0.97); *p*-value: 0.03], EFS [HR: 0.79 (95% CI 0.66–0.94); *p*-value: 9.01e-03], HIF3A [HR: 0.68 (95% CI 0.56–0.84); *p*-value: 2.84e-04], WFDC2 [HR: 0.83 (95% CI 0.74–0.94); *p*-value: 2.52e-03]}; same observation was found with reference to DFS in WFDC2 [HR: 0.79 (95% CI 0.64–0.98); *p*-value: 0.03], HIF3A [HR: 0.70 (95% CI 0.49–0.99); *p*-value: 0.04], HFE [HR: 0.55 (95% CI 0.36–0.84); *p*-value: 5.34e-03], FAXDC2 [HR: 0.57 (95% CI 0.38–0.86); *p*-value: 7.66e-03], CYP27A1 [HR: 0.64 (95% CI 0.47–0.87); *p*-value: 4.19e-03], C8orf88 [HR: 0.45 (95% CI 0.27–0.76); *p*-value: 2.67e-03], and C2orf88 [HR: 0.56 (95% CI 0.37–0.85); *p*-value: 5.84e-03] (Figure 7B). Next, we analyzed the expression levels of these driver genes in GSE21034, which indicated that GSTM2, HAAO, C2orf88, CYP27A1, FAXDC2, HFE, GSTP1, EFS, and WFDC2 were all downregulated in PCa (Figure 8A). C8orf88 was not found in the GSE21034 dataset. The Human Protein Atlas was further utilized to explore the protein levels of driver genes, which suggested that GSTM2, HAAO, C2orf88, CYP27A1, HFE, C8orf88, GSTP1, EFS, HIF3A, and WFDC2 showed varying degrees of lower expression in PCa (Figure 8B). FAXDC2 is nonexistent in PCa in the Human Protein Atlas.

### Exploration of the Correlations of Driver Genes With Unique Characteristics in Prostate Cancer

**FIGURE 9:**
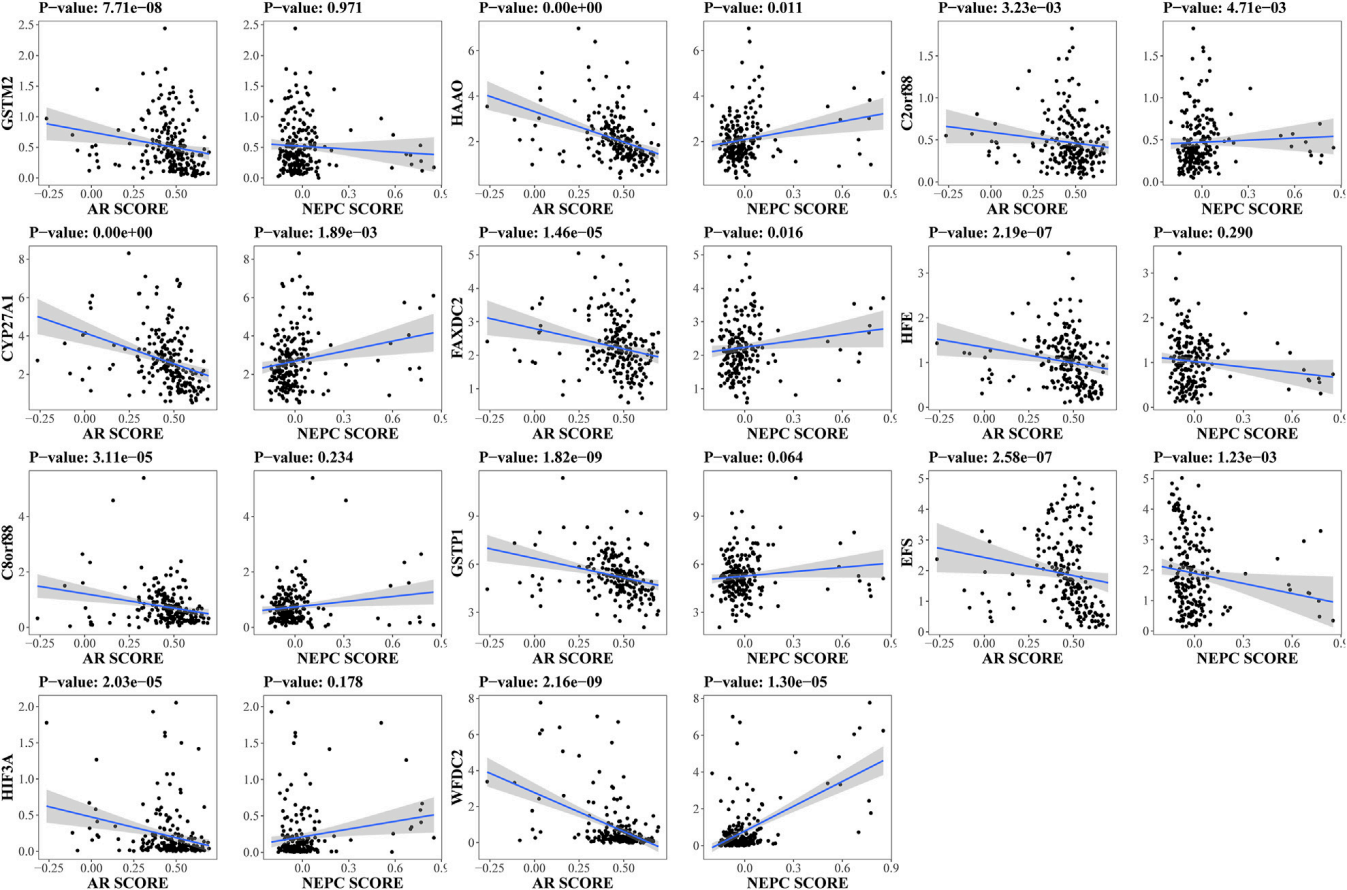
Exploration of correlation between expression levels of driver genes and unique characteristics of PCa. Spearman correlation analysis was conducted in SU2C dataset to disclose their associations. AR score: Androgen receptor score; NEPC score: Neuroendocrine prostate cancer score.

**FIGURE 10:**
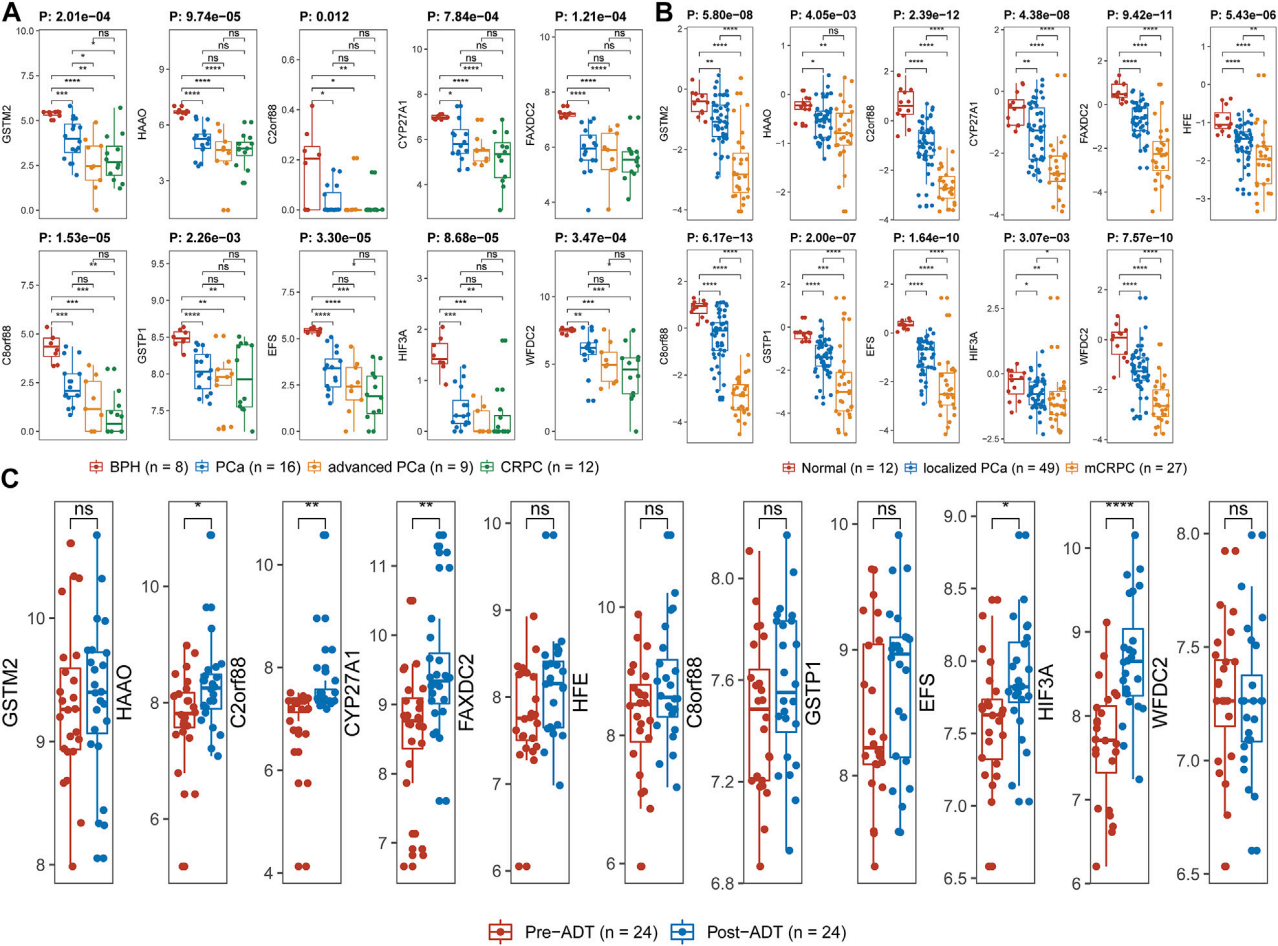
Exploration of expression levels of driver genes across varied stages of PCa. **(A)** GSE80609, **(B)** GSE35988, and **(C)** GSE111177. Wilcoxon rank-sum test was applied between two groups. Kruskal–Wallis rank-sum test was used for more than two groups. Label (*) means *p* < 0.05, label (**) means *p* < 0.01, and label (***) means *p* < 0.001.

By reason of the observed correlation between aneuploidy and ANDROGEN_RESPONSE (Figure 4), we explored the associations between AR scores and driver genes, which indicated that all driver genes were highly correlated with AR score [GSTM2 (Cor: −0.36; *p*-value: 7.71e-08), HAAO (Cor: −0.50; *p*-value <2.2e-16), C2orf88 (Cor: −0.20; *p*-value: 3.23e-03), CYP27A1 (Cor: −0.47; *p*-value <2.2e-16), FAXDC2 (Cor: −0.30; *p*-value: 1.46e-05), HFE (Cor: −0.35; *p*-value: 2.19e-07), C8orf88 (Cor: −0.29; *p*-value: 3.11e-05), GSTP1 (Cor: −0.40; *p*-value: 1.82e-09), EFS (Cor: −0.35; *p*-value: 2.58e-07), HIF3A (Cor: −0.29; *p*-value: 2.03e-05), and WFDC2 (Cor: −0.40; *p*-value: 2.16e-09)] (Figure 9). Additionally, HAAO, C2orf88, CYP27A1, FAXDC2, EFS, and WFDC2 also showed correlation with NEPC scores (Figure 9). As PCa in TCGA mainly referred to localized PCa, we further analyzed the expression levels of these driver genes across varying stages of PCa in the GSE80609 and GSE35988 datasets. GSE80609 suggested insignificant difference of all driver genes between advanced PCa and CRPC (Figure 10A). Nevertheless, when comparing CRPC with localized PCa, all driver genes except HAAO held statistical difference (Figure 10B). Due to the correlation between driver genes and AR-signaling we observed (Figure 5F), we implemented the Wilcoxon rank sum test and found significant expression difference of HAAO, C2orf88, CYP27A1, EFS, and HIF3A between pre-ADT and post-ADT (Figure 10C).

## Discussion

PCa, the most common cancer and the second leading cause of cancer deaths in men in the United States, contributed to a total of an estimated 191,930 new cases and 33,330 deaths in 2020 (Siegel et al., 2020). Emerging pieces of evidence indicated that aneuploidy played a vital role in the progression of PCa. Here, we conducted a systematic and comprehensive analysis of aneuploidy in mutation profile, methylation profile, and gene expression profile.

In the current study, we estimated AS for 459 PCa samples (Figure 2); univariable Cox analysis and Kaplan–Meier analysis suggested that aneuploidy was correlated with prognosis of PCa (Figures 3C,D). Besides, aneuploidy was found to be implicated in the progression and metastasis of PCa (Figure 3E; Table 1), as many studies have indicated (20–23).

GSEA revealed that the number of biological processes in which aneuploidy was involved showed an increasing trend from mutation profile to expression profile, which implied that the effects of aneuploidy accumulated and eventually led to the dysregulation of numerous process pathways (Figure 4). The high enrichment of the proliferation-related process (E2F_TARGETS, G2M_CHECKPOINT, MITOTIC_SPINDLE, P53_PATHWAY, and MYC_TARGETS_V1) also confirmed the findings mentioned earlier that aneuploidy was associated with the progression and metastasis of PCa. Among these pathways, the E2F_TARGETS pathway involves varying cell cycle progression, including regulation of DNA replication and mitosis, DNA damage repair, and differentiation and apoptosis (Dimova and Dyson, 2005; Bracken et al., 2004; Polager and Ginsberg, 2008)–(Dimova and Dyson, 2005; Bracken et al., 2004; Polager and Ginsberg, 2008). G2M_CHECKPOINT and MITOTIC_SPINDLE both were the reflections of mitosis; the former refers to entry into mitosis and has been reported to be associated with chromosome instability; the latter mainly performs the role of segregation of chromosome in cell division; both of which have intrinsic connections with aneuploidy (Löbrich and Jeggo, 2007; Mistry and Oh, 2013). P53, the protein of the TP53 gene, acts as a tumor suppressor and plays vital importance in the cell cycle (Lane and Levine, 2010). The P53 pathway has been indicated to implicate the development and metastasis of PCa (Zhang et al., 2020a; He et al., 2019). Numerous pieces of evidence suggested that MYC_TARGETS_V1 was also associated with cell cycle progression and referred to the transformation of carcinoma (Boxer and Dang, 2001; Grandori et al., 2000; Kim et al., 2008; Grandori et al., 2000; Boxer and Dang, 2001; Kim et al., 2008). These all suggested that aneuploidy was highly implicated in the progression and metastasis of PCa.

Next, we comprehensively and systematically estimated the aneuploidy-related genes in mutation, DNA methylation, and gene expression profile. Gene mutations associated with aneuploidy is limited; only TP53 presented a significant correlation with aneuploidy, which has been validated in previous studies (Ciriello et al., 2013; Zack et al., 2013; Davoli et al., 2017); nevertheless, we did not find any recurrent mutation related to aneuploidy in PCa. Further methylation analysis and gene expression analysis refined 11 driver genes (GSTM2, HAAO, C2orf88, CYP27A1, FAXDC2, HFE, C8orf88, GSTP1, EFS, HIF3A, and WFDC2). GSEA validated 11 genes greatly implicated in cell cycle pathway and proliferation-related pathways (MYC_TARGETS_V1, E2F_TARGETS, and G2M_CHECKPOINT) in PCa.

With regard to these 11 driver genes (GSTM2, HAAO, C2orf88, CYP27A1, FAXDC2, HFE, C8orf88, GSTP1, EFS, HIF3A, and WFDC2), all of which were hyper-methylated and hypo-expressed in PCa; Cox analysis confirmed their prognostic implications (except GSTM2). GSE21034 and the Human Protein Atlas confirmed their differential expression between PCa and normal control samples (Figure 8). SU2C further indicated that all driver genes were associated with AR score (Figure 9), so we explored the expression difference between pre-ADT and post-ADT; we found that HAAO, C2orf88, CYP27A1, EFS, and HIF3A were highly related to ADT (Figure 10C). We also explored the expression levels of 11 driver genes among varied stages of PCa; the Wilcoxon rank-sum test suggested that most of them presented the significant difference between advanced PCa (including CRPC) and localized PCa, which implied they were involved in the progression and development of PCa. These all suggested that these genes contributed to the progression of PCa.

Several studies about GSTM2 have been conducted in PCa. Albawardi et al. (2021) found that GSTM2 was amplificated in PCa based on SNP microarrays. In addition, three previous studies showed that GSTM2 was hyper-methylated in PCa (Patel et al., 2019; Angulo et al., 2016; Ashour et al., 2014; Patel et al., 2019; Angulo et al., 2016; Ashour et al., 2014), which was consistent with our findings. It was also suggested that methylation of GSTM2 significantly correlated with the prognosis of PCa (Angulo et al., 2016). HAAO, which has been reported as the specific methylation biomarker for PCa, presented accurate discrimination between PCa and normal control samples and significantly prognostic implications (Li et al., 2021; Litovkin et al., 2014; Mahapatra et al., 2012; Li et al., 2021; Litovkin et al., 2014; Mahapatra et al., 2012). CYP27A1, which is a P450 enzyme catalyzing androgen-anabolism, was suggested to be regulated by both genetic and epigenetic processes in PCa (Maksymchuk and Kashuba, 2020). Increasing pieces of evidence suggested that CYP27A1 was downregulated in PCa that was confirmed in both messenger RNA and protein levels and was associated with the progression of PCa (Alfaqih et al., 2017; Dambal et al., 2020; Khan et al., 2019). Tamura et al. (2007) indicated that CYP27A1 was downregulated, comparing hormone-refractory PCa with hormone-sensitive PCa. Numerous studies confirmed the prognostic implication of CYP27A1 in PCa (Maksymchuk and Kashuba, 2020; Alfaqih et al., 2017). Although that the downregulation of CYP27A1 in PCa contributed to the progression of PCa has been widely reported, the underlying association of CYP27A1 with aneuploidy was firstly confirmed in the current study. Paradoxically, downregulated expression levels of HFE in PC3 (PCa cells) have been validated to inhibit the development and metastasis of PC3 cells (Lin et al., 2017). This suggested that our exploration of the impact of HFE on PCa, both *in vivo* or *in vitro*, was in demand. The hyper-methylation of promoter and upregulation of expression levels of GSTP1 in PCa has also been widely reported (Zavridou et al., 2021; Qiu et al., 2020; Wang et al., 2020; Zhang et al., 2020b; Fatma Karaman et al., 2019); more than that, the methylation of GSTP1 has been identified as the specific and accurate biomarker for the diagnosis of PCa (Guo et al., 2020; Constâncio et al., 2019; Fiano et al., 2019). Zavridou et al. (2021) revealed that the hyper-methylation of promoter and the upregulation of the expression levels of GSTP1 existed in circulating tumor cells and exosomes of mCRPC and was associated with the overall survival of PCa. Mohammadi et al. (2020) indicated that GSTP1 was upregulated by hormone therapy, although we did not get a difference of GSTP1 (Figure 10C), which may be due to lack of sufficient samples. Three previous studies indicated that EFS was significantly downregulated in PCa owing to DNA methylation and was proven to be a tumor suppressor gene in PCa (Wang et al., 2020; Sertkaya et al., 2015; Vanaja et al., 2009). Consistent with our study, HIF3A and WFDC2 were validated to be hyper-methylated and downregulated in PCa (Bjerre et al., 2019; Geybels et al., 2015; Xiong et al., 2020; Kim et al., 2011; Kim et al., 2011; Geybels et al., 2015; Bjerre et al., 2019; Xiong et al., 2020). HIF3A was also suggested to associate with the prognosis of PCa and could be regarded as a diagnostic biomarker for PCa with an area under the receiver operating characteristic curve of more than 0.99 (Mahapatra et al., 2012; Bjerre et al., 2019), and WFDC2 implicated the development and metastasis of PCa by regulating EGFR signaling pathway (109). Few studies have been conducted on C2orf88, C8orf88, and FAXDC2; we first uncovered the association of them with aneuploidy in PCa.

Nevertheless, there remain some limitations in our study, other experimental validations for our findings are in demand, and the detailed molecular mechanism for these driver genes has not been investigated; therefore, further efforts on the exact molecular mechanism of GSTM2, HAAO, C2orf88, CYP27A1, FAXDC2, HFE, C8orf88, GSTP1, EFS, HIF3A, and WFDC2 both *in vitro* and *in vivo* are required.

In conclusion, we systematically demonstrated the molecular process of aneuploidy in PCa and identified 11 potential driver genes (GSTM2, HAAO, C2orf88, CYP27A1, FAXDC2, HFE, C8orf88, GSTP1, EFS, HIF3A, and WFDC2). Our findings could shed light on the tumorigenesis of PCa and provide a better understanding of the development and metastasis of PCa; in addition, all of them could be promising and actionable therapeutic targets pointing to aneuploidy.

## Data Availability

Publicly available datasets were analyzed in this study. These data can be found here: TCGA database (http://portal.gdc.cancer.gov/), GTEx database (http://www.gtexportal.org/), GEO database (http://www.ncbi.nlm.nih.gov/geo/), and cBioPortal database (https://www.cbioportal.org/).
