# Supplementary figures and images for: Systematic Elucidation of the Aneuploidy Landscape and Identification of Aneuploidy Driver Genes in Prostate Cancer
Source: Front Cell Dev Biol. 2022 Jan 21;9:723466. doi: 10.3389/fcell.2021.723466 (PMC8814427; doi:10.3389/fcell.2021.723466)

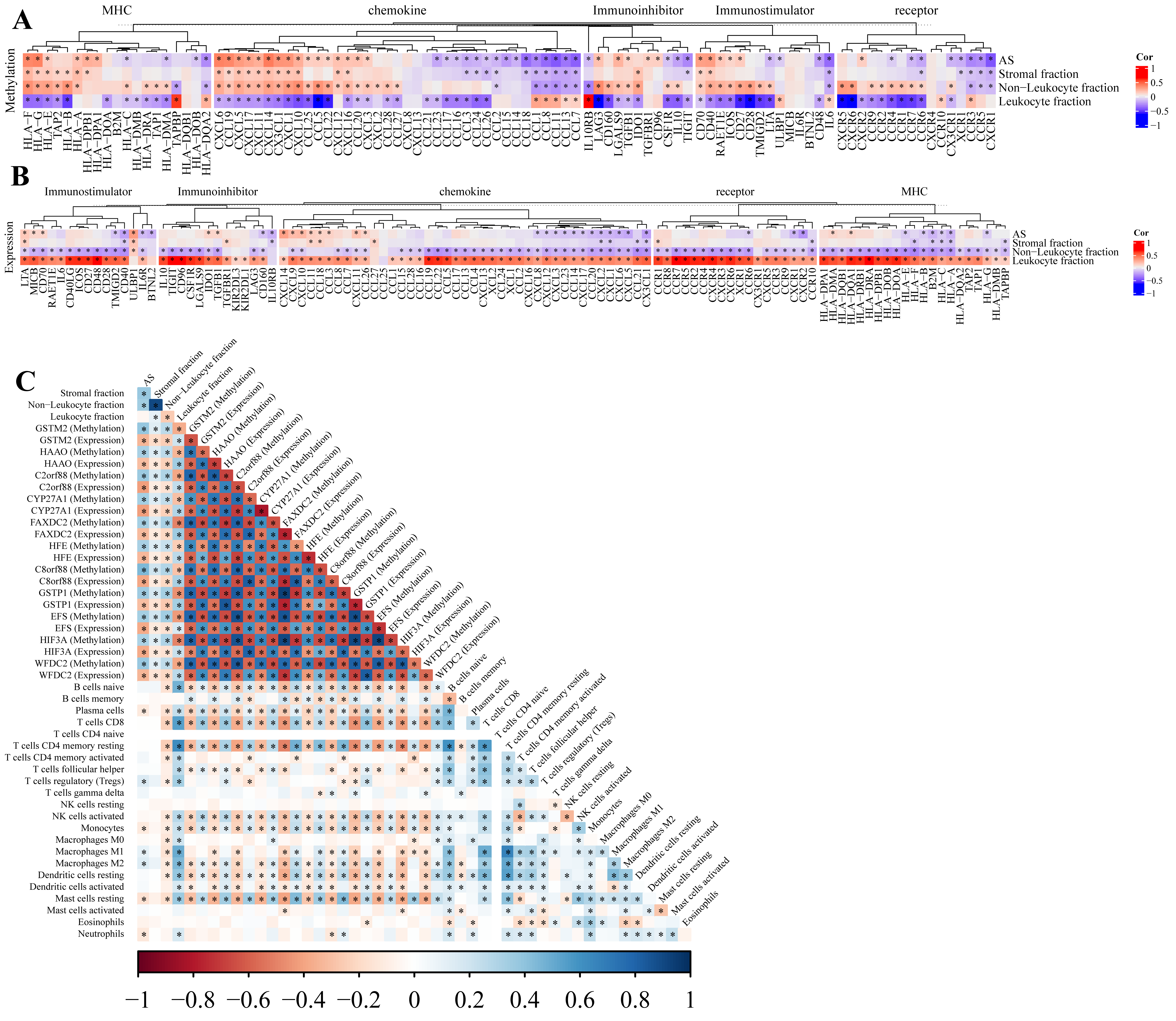

Supplement: Supplementary file 1 [file Image6.TIF]

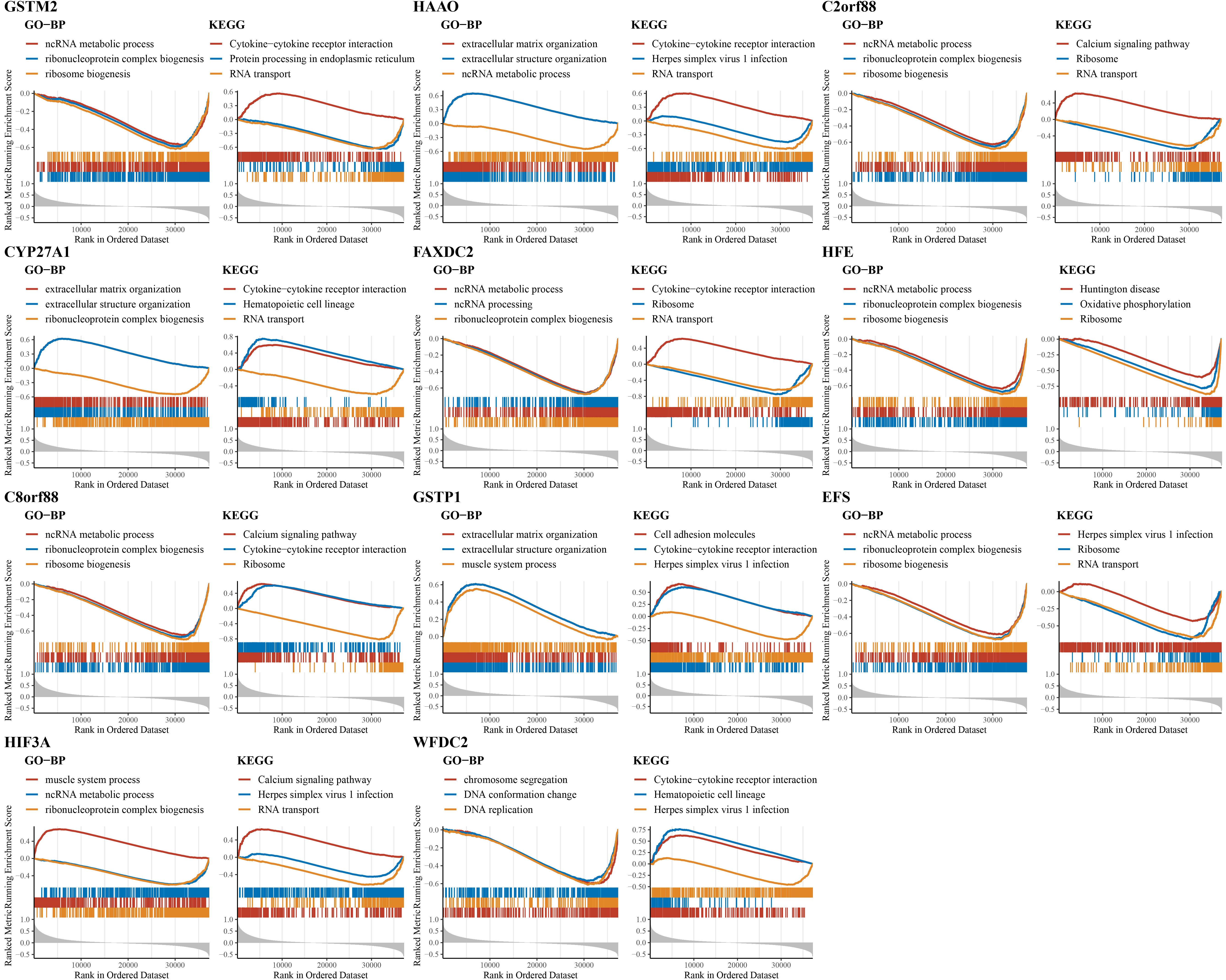

Supplement: Supplementary file 2 [file Image3.TIF]

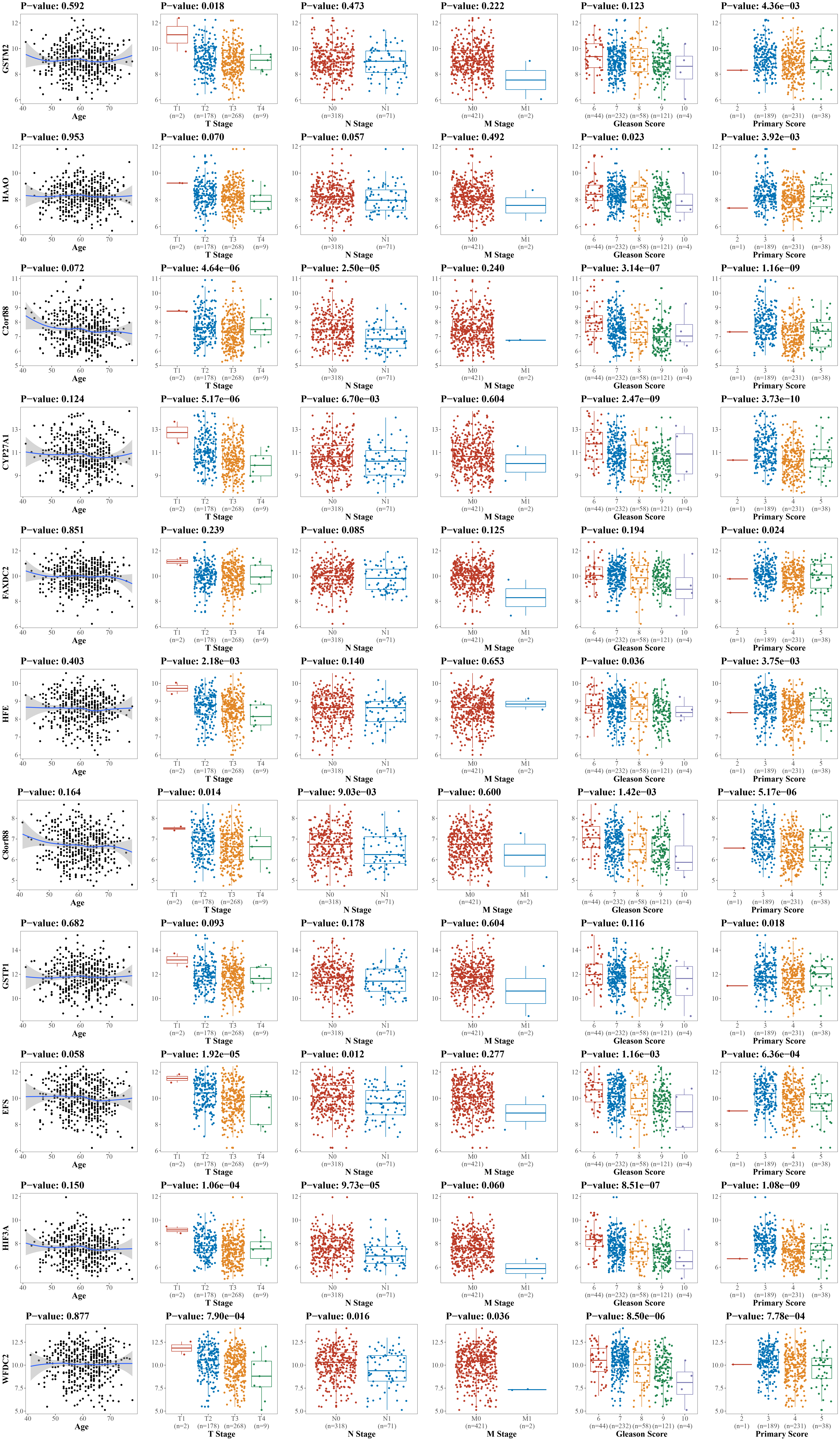

Supplement: Supplementary file 3 [file Image4.TIF]

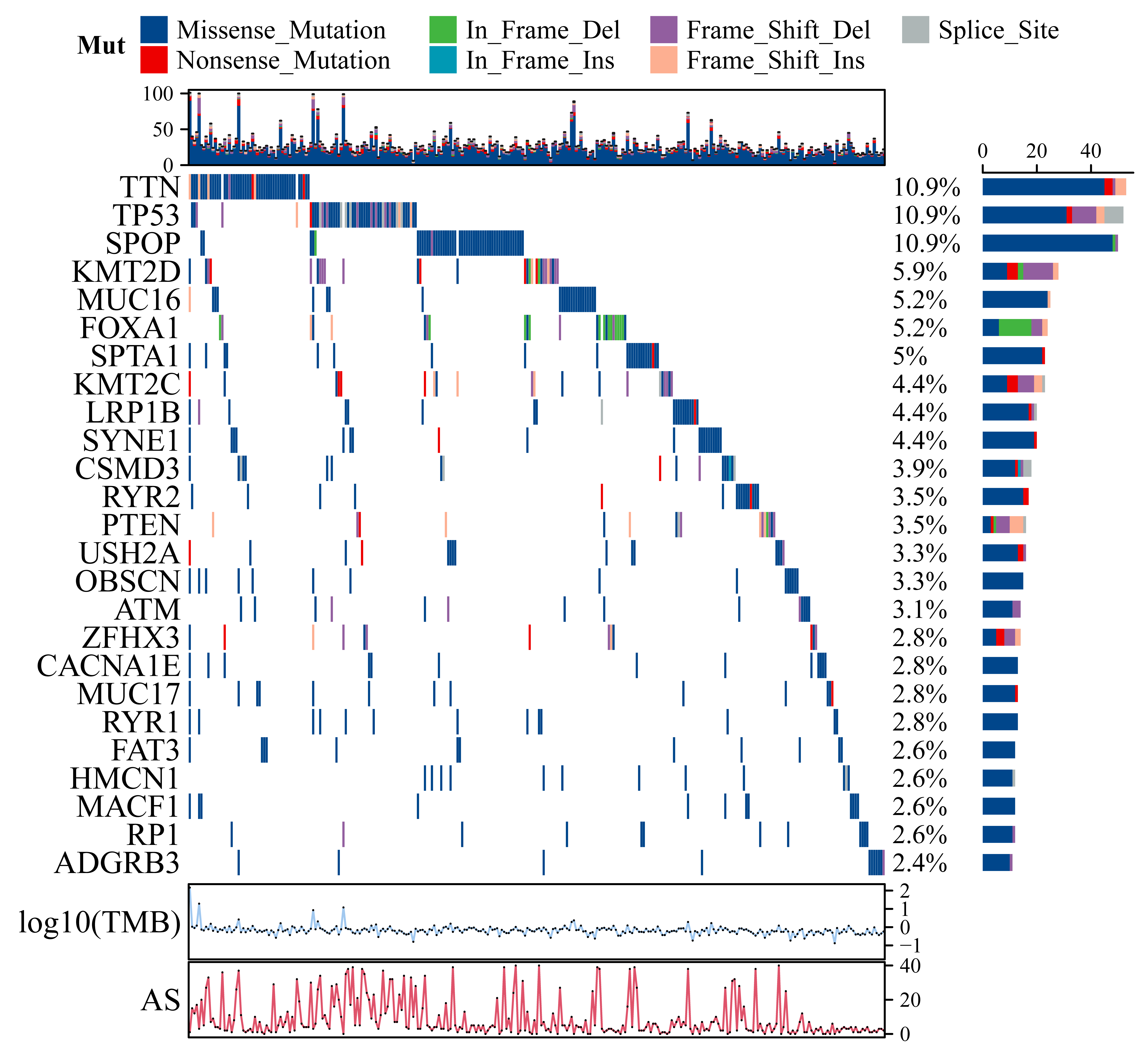

Supplement: Supplementary file 4 [file Image2.TIF]

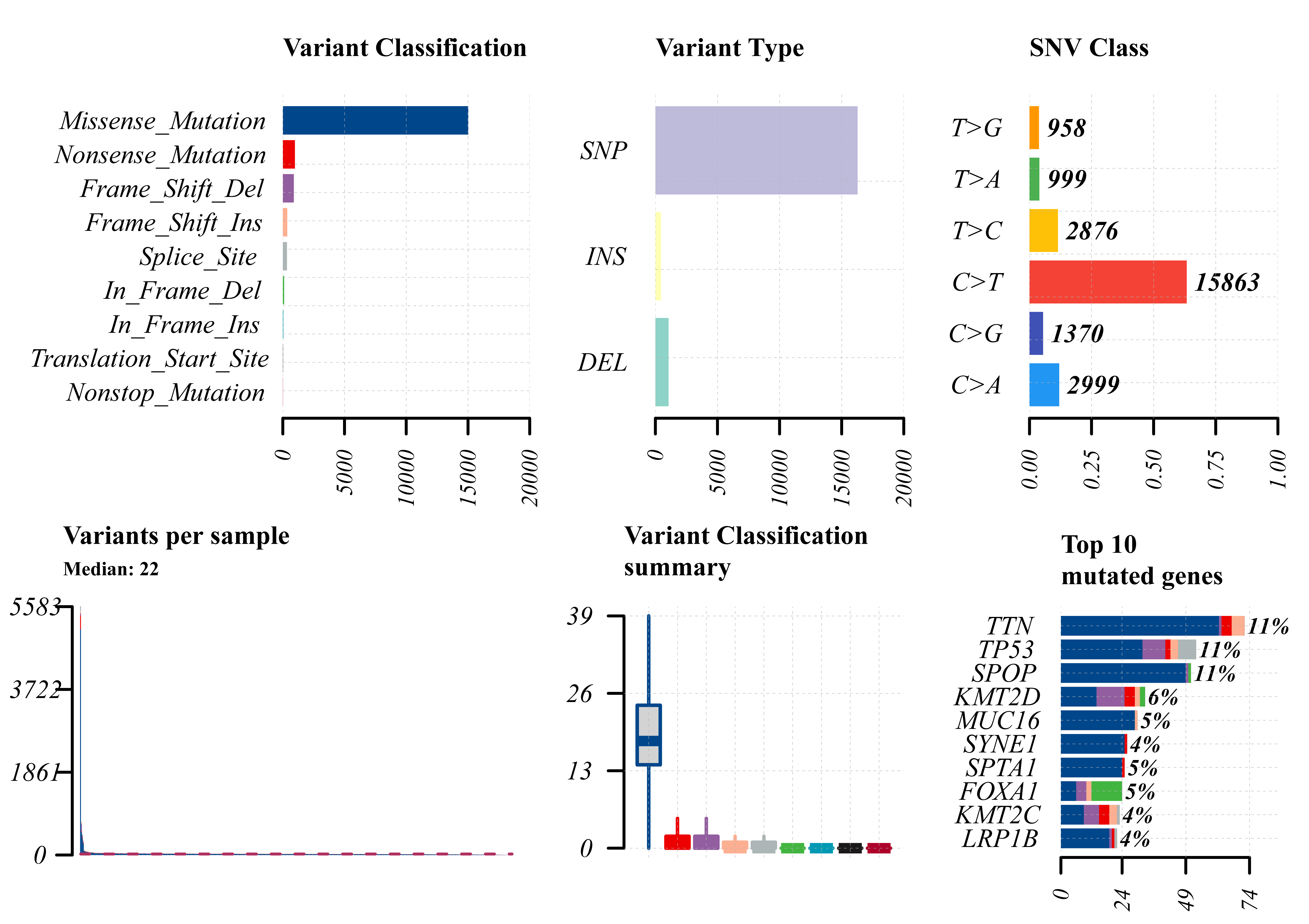

Supplement: Supplementary file 5 [file Image1.TIF]

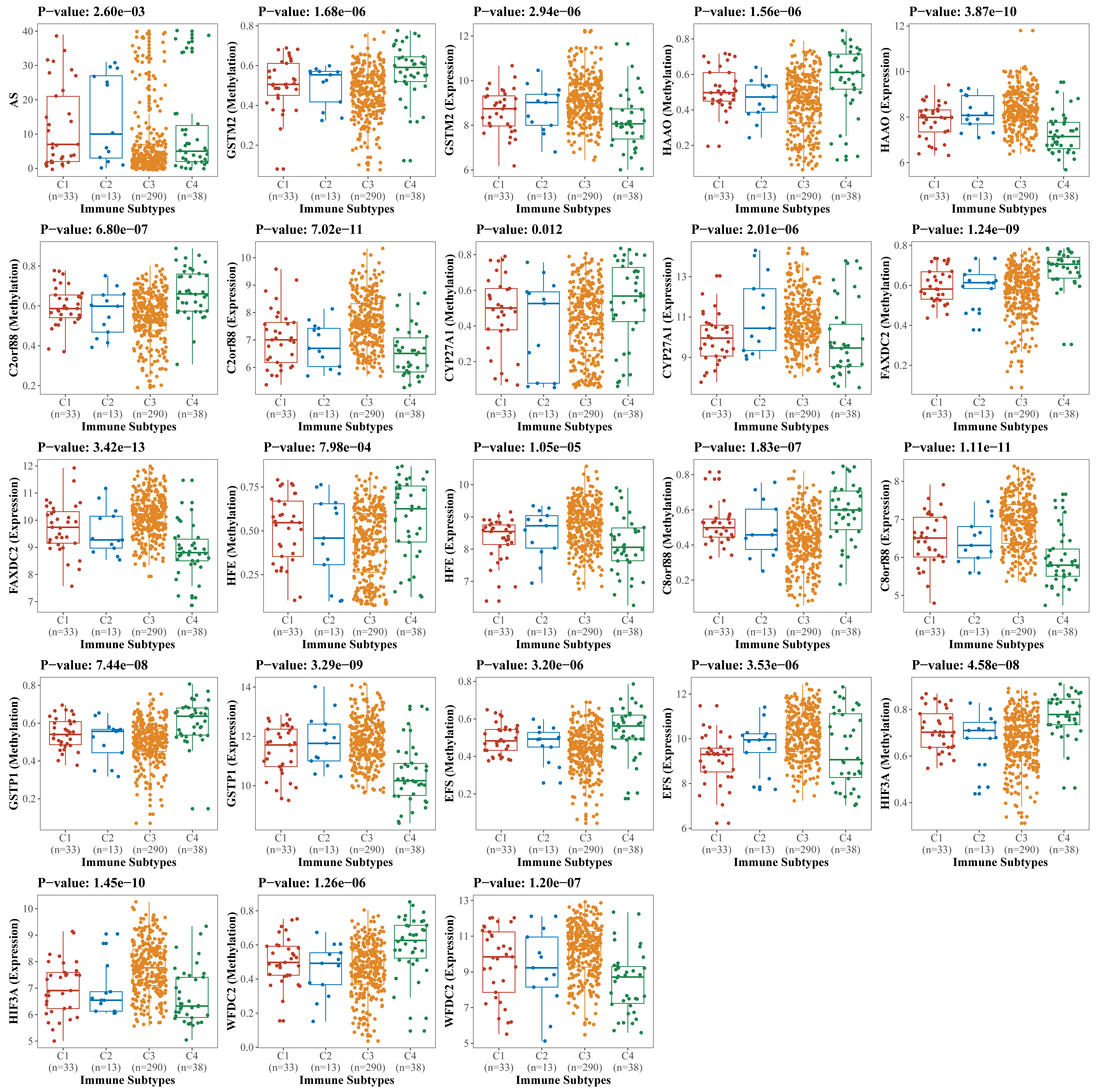

Supplement: Supplementary file 6 [file Image5.TIF]
